# Supplementary material for: Effectiveness of a pharmacist diabetes coaching program: A propensity-matched retrospective analysis
Source: PLoS One. 2026 Mar 20;21(3):e0345534. doi: 10.1371/journal.pone.0345534 (PMC13004327; doi:10.1371/journal.pone.0345534)
Supplement: Attachment — Submitted filename: DCC.ResponseToReviewers.1.14.26.docx [file pone.0345534.s002.docx]

Response to Reviewers

We appreciate the thoughtful and constructive feedback provided by the Academic Editor and reviewers. We have carefully considered all comments and revised the manuscript accordingly. The table below presents a point-by-point response to each comment, including the verbatim comment, our response, a description of the revisions made, and the location(s) of these revisions in the manuscript. Changes are highlighted in the accompanying tracked-changes version.

Table of Reviewer Comments with Responses and Corresponding Revisions.

| Reviewer # | Comment # | Reviewer Comment (Verbatim) | Response to Comment | Revisions Made | Location of Revisions Made |
| --- | --- | --- | --- | --- | --- |
| Academic Editor | 1 | Please state what role the funders took in the study. | This study was conducted as a pragmatic, internally supported clinical initiative rather than an externally funded research project. Operational support for the diabetes coaching program was provided by the UMMH Diabetes Center and Shields Health as part of routine care delivery. | Added Role of Funders statement using required PLOS ONE language. | Cover Letter |
| Academic Editor | 2 | Funding information should not appear in any section or other areas of your manuscript. | All references to funding have been removed from the manuscript text. Operational costs associated with delivery of the diabetes coaching program are described only within the cost analyses and are not presented as funding information, in accordance with PLOS ONE policy. | Removed funding language from manuscript body. | Acknowledgements |
| Academic Editor | 3 | Data Availability Statement is missing contact details for the third party. | We updated the Data Availability Statement to explain ethical and legal restrictions on data sharing and to include contact information for data access requests. | Revised Data Availability Statement. | Data Availability Statement |
| Academic Editor | 4 | Please upload a new copy of Figure 1 as the detail is not clear. | We uploaded a revised high-resolution version of Figure 1 using the Picture Analysis and Conversion Engine. | Uploaded Figure 1 with high-resolution version. | Figure 1 |
| Academic Editor | 5 | Please ensure that you refer to Figures 2 and 3 in your text. | We have added references to Figures 2 and 3 in the Results section. | Added figure citations in text. | Results |
| Reviewer #1 | 1 | Because participation in the program was voluntary rather than randomized, there is a significant risk of selection bias. | We agree and expanded the Limitations section to explicitly discuss voluntary enrollment, potential selection bias, unmeasured confounding, and implications for interpretation. | Expanded limitations discussion. | Discussion – Limitations |
| Reviewer #1 | 2 | The manuscript does not specify how many pharmacists served as coaches, their full-time equivalency, or the patient-to-coach staffing ratio. | We expanded the Methods section to report the number of pharmacists (2) and patient-to-coach ratios. | Added staffing and workforce description in intervention description. | Methods – Setting |
| Reviewer #1 | 3 | The manuscript does not provide information about the content, duration, credentialing, or cost of training for pharmacist coaches. | We added details describing pharmacist training, credentialing, duration, and clarified that training costs were not included in the financial analysis. | Added training description and cost clarification. | Methods – Setting |
| Reviewer #1 | 4 | The frequency, duration, and structure of telehealth encounters are not reported. | We added details regarding typical encounter frequency, duration, and modalities to improve reproducibility. | Added encounter structure description. | Methods – Setting |
| Reviewer #1 | 5 | The manuscript would benefit from reporting the total number of diabetes patients served to contextualize the sample. | We added contextual information describing the overall diabetes population served by the UMMH Diabetes Center during the study period. | Added population context. | Methods – Setting |
| Reviewer #1 | 6 | A brief justification for selecting pharmacists rather than nurses or physicians would improve clarity. | We added a justification highlighting pharmacists’ expertise in diabetes pharmacotherapy, medication titration, and integration with specialty pharmacy workflows. | Added rationale for pharmacist-led model. | Methods – Setting |
| Reviewer #2 | 1 | I would suggest a table demonstrating changes in glycemic control, health care utilization, and total medical expenditures. | We added a table summarizing pre, post, and change data on glycemic control, health care utilization, and TME to complement the narrative results. | Added Table 3. | Results – Table 3 |
| Reviewer #2 | 2 | Can the authors clarify how they adjusted for COVID during the study period? | We clarified how the difference-in-differences design accounted for COVID-related secular trends and acknowledged residual effects as a potential limitation. | Added COVID clarification and limitation. | Methods; Discussion – Limitations |
