# Supplementary material for: Effectiveness of a pharmacist diabetes coaching program: A propensity-matched retrospective analysis
Source: PLoS One. 2026 Mar 20;21(3):e0345534. doi: 10.1371/journal.pone.0345534 (PMC13004327; doi:10.1371/journal.pone.0345534)
Supplement: Attachment — Submitted filename: DCCevaluation.Resub2.ResponseToReviewers.2.12.26.docx [file pone.0345534.s003.docx]

Response to Reviewers

We appreciate the thoughtful and constructive feedback provided by the Academic Editor and reviewers. We have considered all comments and revised the manuscript accordingly. The table below presents a point-by-point response to each comment, including the verbatim comment, our response, a description of the revisions made, and the location(s) of these revisions in the manuscript. Changes are highlighted in the accompanying tracked-changes version.

Table of Reviewer Comments with Responses and Corresponding Revisions.

| Reviewer # | Comment # | Reviewer Comment (Summarized) | Response to Comment | Revisions Made | Location of Revisions Made |
| --- | --- | --- | --- | --- | --- |
| Reviewer 1 | 1 | Consider explicitly reporting the number of pharmacist coaches involved in the program, the full-time equivalent staffing dedicated to the intervention, and the resulting patient-to-coach ratio. | We added additional information on staffing structure and capacity. | Reported number of coaches (n=2), total program effort (2.0 FTE), and approximate patient-to-coach ratio (80:1 at capacity). | Methods – Setting Paragraph 2 |
| Reviewer 1 | 2 | Provide additional information on pharmacist training. Describe the content, duration, credentialing, and approximate cost of the additional diabetes-related training received by the pharmacists, and clarify whether training costs were included in the financial analysis. | We expanded the training description and clarified that DSMES/CDCES development was part of clinical professional growth rather than formal intervention implementation, and therefore excluded from financial analysis. | Added onboarding training details, DSMES exposure, CDCES credential attainment, and explicit statement that training costs were not included in financial analysis. | Methods – Setting Paragraph 2 |
| Reviewer 1 | 3 | Provide greater clarity regarding telehealth delivery, including frequency, duration, and structure of encounters. | We clarified the telehealth structure and emphasized flexibility to reflect real-world implementation rather than a rigid protocol. | Added visit cadence (weekly then biweekly), visit duration, structure of encounters, and statement that the intervention was intentionally flexible. | Methods – Setting Paragraph 3 |
| Reviewer 1 | 4 | If available, summarize telehealth contact distribution or program dose. | We clarified typical encounter structure and frequency and described the flexible delivery model used in practice. | Added description of typical telehealth cadence and clinical tailoring approach. | Methods – Setting Paragraph 3 |
| Reviewer 1 | 5 | Frame outcomes carefully given non-significant findings. | We revised the Abstract Conclusions and Discussion to emphasize directional trends, exploratory interpretation, and pragmatic real-world context rather than confirmatory conclusions. | Reworded Abstract and Discussion language to reflect directional findings, pragmatic interpretation, and consistency of trends. | Abstract – Conclusions; Discussion |
